# Real-world data on prognosis and outcome of primary plasma cell leukemia in the era of novel agents: a multicenter national study by the Greek Myeloma Study Group

**DOI:** 10.1038/s41408-018-0059-6

**Published:** 2018-03-09

**Authors:** Eirini Katodritou, Evangelos Terpos, Sossana Delimpasi, Maria Kotsopoulou, Eurydiki Michalis, Chrysanthi Vadikolia, Marie-Christine Kyrtsonis, Argiris Symeonidis, Nikolaos Giannakoulas, Chrissa Vadikolia, Michalis Michael, Christina Kalpadakis, Theodora Gougopoulou, Chrystalla Prokopiou, Georgia Kaiafa, Dimitrios Christoulas, Maria Gavriatopoulou, Evlampia Giannopoulou, Vasiliki Labropoulou, Evgenia Verrou, Efstathios Kastritis, Pavlina Konstantinidou, Achilles Anagnostopoulos, Meletios A. Dimopoulos

**Affiliations:** 1 Department of Hematology, Theagenio Cancer Hospital, Thessaloniki, Greece; 2 Department of Clinical Therapeutics, School of Medicine, National and Kapodistrian University of Athens, Athens, Greece; 3 Department of Hematology and Bone Marrow Transplantation Unit, Evangelismos Hospital, Athens, Greece; 4 Department of Haematology, Metaxa Cancer Hospital, Piraeus, Greece; 5 Department of Hematology, “G. Gennimatas” General Hospital, Athens, Greece; 6 Department of Hematology and Bone Marrow Transplantation Unit, George Papanikolaou Hospital, Thessaloniki, Greece; 7 First Department of Propaedeutic Internal Medicine, Laikon General Hospital, National and Kapodistrian University of Athens, Athens, Greece; 8 Department of Internal Medicine, Division of Hematology, University of Patras Medical School, Patras, Greece; 9 Department of Hematology, School of Medicine, University of Thessaly, Larisa, Greece; 10 Department of Hematology, 424 General Military Hospital, Thessaloniki, Greece; 11 Department of Hematology, Nicosia General Hospital, Nicosia, Cyprus; 12 Department of Hematology, School of Medicine, University of Crete, Heraklion, Greece; 13 Department of Hematology, University of Ioannina School of Medicine, Ioannina, Greece; 14 Department of Hematology, New General Limassol Hospital, Limassol, Cyprus; 15 Hematology Department, AHEPA University Hospital, Thessaloniki, Greece; 16 Department of Hematology, 251 General Air Force Hospital, Athens, Greece

## Abstract

We have studied the efficacy and the prognostic impact of novel agents in 50 primary plasma cell leukemia (pPCL) patients registered in our database. Eighty percent of patients were treated upfront with novel agent-based combinations; 40% underwent autologous stem cell transplantation (ASCT). Objective response rate was 76; 38% achieved at least very good partial response (≥vgPR) and this correlated significantly with bortezomib-based therapy plus ASCT. At the time of evaluation, 40 patients had died. Early mortality rate (≤1 month) was 6%. Median progression-free survival (PFS) and overall survival (OS) were 12 months and 18 months respectively, both significantly longer in patients treated with bortezomib-based therapy + ASCT vs. others (PFS: 18 vs. 9 months; *p* = 0.004, OS: 48 vs. 14 months; p = 0.007). Bortezomib-based therapy + ASCT predicted for OS in univariate analysis. In multivariate analysis, achievement of ≥vgPR and LDH ≥ 300 U/L were significant predictors for OS. These real-world data, based on one of the largest reported national multicenter series of pPCL patients treated mostly with novel agents support that, among the currently approved induction therapies, bortezomib-based regimens are highly effective and reduce the rate of early mortality whereas in combination with ASCT consolidation they prolong OS.

## Introduction

Primary plasma cell leukemia (pPCL), is a distinct clinicopathological entity of plasma cell dyscrasias accounting for about 60–70% of all plasma cell leukemia cases^1,2^. Diagnosis of pPCL requires both 2 × 10^3^/μL peripheral blood clonal plasma cells and plasmacytosis accounting for >20% of the differential white cell count^3^, though, in some studies, it was considered sufficient to meet only one of these two diagnostic criteria^4^. The definition of pPCL is arbitrary and a lower diagnostic threshold (i.e., 5% and/or >0.5 × 10^3^/μL) has been recently proposed^4^. Primary PCL does not arise from pre-existing multiple myeloma (MM) but it is presented as de novo disease^5^ and it is characterized by an aggressive clinical course^4,5^. In the past, the only available treatment for patients with pPCL was conventional chemotherapy which failed to control the disease leading to dramatically poor outcome^6–8^. During the last decades, initially autologous stem cell transplantation^9^ (ASCT) and subsequently novel therapies^10^ including immunomodulatory drugs and proteasome inhibitors (PIs) namely bortezomib, led to a slight improvement of pPCL patients’ survival^5^. The efficacy of bortezomib in pPCL has been demonstrated by our group and others in small retrospective series^5,11–13^, however, this has been questioned in other studies^14,15^. Recently, a prospective phase 2 study has demonstrated that induction therapy with bortezomib based combinations followed by ASCT led to high response rates and prolonged survival in patients with pPCL^16^. Bortezomib based therapies are currently recommended for the management of pPCL^4^ however, optimal treatment, remains an unmet clinical need and allogeneic transplantation, is the only therapeutic approach that is capable to rescue at least a limited number of young and fit patients^9^.

Considering the rarity of pPCL, data regarding response to therapies, prognostication and outcome of pPCL patients in the real-world setting are limited. Therefore, the purpose of this study was to validate the efficacy and prognostic impact of novel agents, mainly bortezomib-based combinations, ASCT and other prognostic factors related to the patient or the disease, in double the number of pPCL patients that we had previously reported^11^. Το our knowledge this is currently one of the largest reported multicenter national study, providing real-world data on prognosis and outcome of an unselected population of pPCL patients, treated in the era of novel agents.

## Subjects and methods

### Patients

We retrospectively studied the medical records of 50 consecutive pPCL patients registered in the Greek Myeloma Study Group (GMSG) database between January 2000 and January 2016 out of 2711 patients with MM. We have chosen as a starting time-point the year 2000, as since then, first generation novel agents were incorporated in the treatment of MM, initially at relapse and subsequently as first line therapy; The term “era of novel agents” is widely used across retrospective studies to describe this period. Primary PCL was defined by the presence of >2 × 10^3^/μL clonal plasma cells in the peripheral blood or plasmacytosis accounting for >20% of the differential white cell count^4^.

### Methodology

The participating physicians were asked to fill in a questionnaire that included clinical and laboratory data of pPCL patients at the time of diagnosis. The required information included, age, gender, performance status according to the Eastern Cooperative Oncology Group scale (ECOG), presence of plasmacytomas at diagnosis, bone disease stage, serum biochemistry, complete blood count, M-protein in serum and/or urine, the proportion of plasma cells in the bone marrow and the peripheral blood, plasma cell immunophenotype, with special emphasis on CD56 expression if available and cytogenetics (including FISH). Additional information included, the date of pPCL diagnosis, the date of relapse, the date of the last follow-up, the survival status of the patients at the time of data recording, as well as information about the type of treatment for pPCL including transplantation, safety data and cause of death. Response to pPCL treatment was evaluated according to the International Myeloma Working Group (IMWG) response criteria^4^. Bortezomib-based regimens were defined as regimens containing bortezomib, administered either once or twice a week at the dose of 1.3–1.5 mg/m2, combined with dexamethasone and other drugs mainly chemotherapy, following the protocols of each center. The study was conducted according to Helsinki Declaration.

### Statistical analysis

Pearson’s *χ*^2^ and Mann–Whitney *U* test were used for correlations. Cox regression Likelihood Ratio univariate and multivariate analysis were used to determine possible independent predictive factors for survival. Progression free survival (PFS) was defined as the time from start of treatment until progression or death whichever comes first. Overall survival (OS) was defined as the time from diagnosis until death from any cause; PFS and OS curves were plotted by using the Kaplan–Meier method and comparisons were performed with the log rank test. Hazard ratios (HzR) were estimated using univariate Cox regression, whereas for the evaluation of the effects of several prognostic factors, a multivariate Cox regression analysis was performed, in which the statistical significance level (*p*-values) was assessed using the Likelihood ratio method. The statistical significance boundary was set to 5%. Data processing and analysis were carried out with the software package SPSS v16.

## Results

### Patients’ characteristics

We analyzed data of 50 consecutive patients with pPCL out of 2711 patients with MM (1.8%) registered in the GMSG database. Male to female ratio was 1:1. The median age was 65.5, (range: 32–86) and it was significantly lower in patients who underwent ASCT after induction treatment vs. those who did not (56 years vs. 67.5; *p* = 0.001). Regarding MM type 19 patients had IgG MM, 9 had IgA MM, 14 patients had light chain myeloma, 2 had IgD MM and in 6 patients MM was defined as non-secretory. Performance status using the ECOG scale was ≥2 in 52% of patients. According to the International staging system (ISS) 29 (58%) patients had advanced disease (ISS3), 16 patients (32%) had ISS2 and only 5 patients (10%) had ISS1; 26% of patients had revised ISS stage (R-ISS) 3; 77% of patients presented with lytic bone disease and 11% with bone or soft tissue plasmacytomas, at diagnosis; 24% of patients had renal insufficiency at diagnosis (eGFR < 40 ml/min/1.73 m^2^). Bence-Jones protein was present in 68% of patients; 53% of patients had abnormal lactate dehydrogenase (LDH); 28% had hypercalcemia and 68% had hemoglobin < 10 g/dL; 89% had β2 microglobulin ≥ 3.5 mg/L; fluorescent in situ hybridization (FISH) or conventional karyotype were available in 32/50 (64%) patients; high risk features were present in 65% of patients; 60% of patients had CD56 (−) peripheral blood monoclonal plasma cells, measured by flowcytometry.

### Treatment and response

Overall, 40 patients received novel agents (80%) at first line; 38 patients (76%) received bortezomib-based combinations and 2 patients received thalidomide-based triplet (melphalan-prednisone-thalidomide); 15/38 patients (40%) treated with bortezomib based combinations and one patient treated with C/T underwent ASCT as part of the induction therapy; median time from induction therapy to ASCT was 4 months (range: 3–7). Treatment regimens during induction are depicted in Table 1; 38/50 patients (76%) achieved objective response (i.e., at least partial response) and 38% displayed at least very good partial response (≥vgPR), including 16% complete responses. Median time to best response was 4 months (range: 2–7) for the whole population; for patients that achieved ≥ vgPR the median time to best response was also 4 months (range: 2–6); response rates according to treatment with bortezomib based regimens ± ASCT or with conventional therapy are demonstrated in Table 2. Regarding non-eligible for transplant patients, bortezomib-based therapies displayed higher efficacy compared to other treatments (Table 2). Achievement of ≥vgPR significantly correlated with bortezomib-based therapy followed by ASCT (*p* = 0.004). In addition, sustained response (i.e., objective response maintained for at least 1 year) significantly correlated with bortezomib-based combination therapy plus ASCT (*p* < 0001).

**Table 1:** Initial therapeutic regimens

Regimen	No. of patients
VCD	19
PAD	8
VDT PACE	4
VDT	2
MPV	2
VD	3
MPT	2
VAD	6
MP	4
ASCT	16
Double ASCT	2
Allo-SCT	1

*VCD* cyclophosphamide, bortezomib, dexamethasone, *VD* bortezomib, dexamethasone, *PAD* bortezomib, adriamycin, dexamethasone, *VDTPACE* bortezomib, dexamethasone, thalidomide, cisplatinum, adriamycin, cyclophosphamide, etoposide, *VDT* bortezomib, dexamethasone, thalidomide, *MPV* melphalan, prednisone, bortezomib, *VD* bortezomib, dexamethasone, *MPT* melphalan, prednisone, thalidomide, *VAD* vincristine, adriamycin, dexamethasone, *MP* melphalan, prednisone, *ASCT* autologous stem-cell transplantation, *Allo-SCT* allogeneic stem cell transplantation

**Table 2:** Response according to treatment

Therapy	Patients, *n*	≥vgPR%	ORR%	vgPR, *n*	CR, *n*	PR, *n*	SD, *n*	PD, *n*
All treatments	50	38	76	11	8	19	3	9
Bortezomib-based, no ASCT	23	26	70	4	2	10	2	5
Bortezomib-based, +ASCT	15	73	100	6	5	4	–	–
Conventional treatment (including 2 MPT)	12	17	58	1	1	5	1	4

*vgPR* very good partial response, *ORR* objective response rate, *CR* complete response, *PR* partial response, *SD* stable disease, *PD* progressive disease, *ASCT* autologous transplantation, *MPT* melphalan, prednisone, thalidomide

### Safety

With regard to grade 3/4 toxicity observed during first-line treatment, 29% of patients experienced neutropenia, 33% had anemia, 27% displayed thrombocytopenia, 18% had gastrointestinal toxicity, and 3% developed peripheral neuropathy; grade 3/4 myelosuppression defined as grade 3/4 toxicity of at least one cell line observed in 32% of patients and significantly correlated with the performance of ASCT (*p* = 0.01); neutropenic infection was observed in 22% of patients, however, none of the patients died from infection during first line treatment. Overall, grade 3/4 toxicity did not correlate with the type of therapy (i.e., conventional vs. novel-agent therapy) and it was in general manageable.

### Outcome

After a median follow up of 18 months (range: 1–100), 80% of patients have died (disease progression: 19, infection: 16, other causes: 5) and 10 patients remain alive. Early mortality (≤1 month) occurred in 3/40 deceased patients and it correlated with treatment without bortezomib (*p* = 0.1).

The median number of treatment lines was 2 (range1–5); 29 patients received second line treatment; among them, 24 patients were treated upfront with bortezomib-based regimens and 5 patients with conventional therapies or MPT (66% vs. 41%; *p* = 0.1); 24/29 (83%) patients were treated in second line with novel agent-combinations: 18/29 (62%) received bortezomib or other PI ± IMID triplets (including 1 patient treated with carfilzomib-pomalidomide-dexamethasone, 1 patient was treated with ixazomib-lenalidomide-dexamethasone and 1 patient was treated with carfilzomib-lenalidomide-dexamethasone) and 7 received LenDex (one plus chemotherapy). Overall, 40/50 patients (80%) received novel agents at first line, and 42/50 patients (84%) received novel agents at any line, including 6 patients receiving next generation novel agents such as carfilzomib, daratumumab, pomalidomide, and ixazomib in different treatment lines. Salvage therapy including novel agents at any line, was offered in both patients treated upfront with bortezomib-based regimens or with conventional therapy (bortezomib-based therapy: 22, conventional therapy: 3 *p* > 0.05).

The median survival after relapse was 6.5 months (range: 0.5–51). Neither second line nor any treatment beyond first line with novel agents positively correlated with OS or post-relapse survival (*p* > 0.05).

**Fig. 1:**
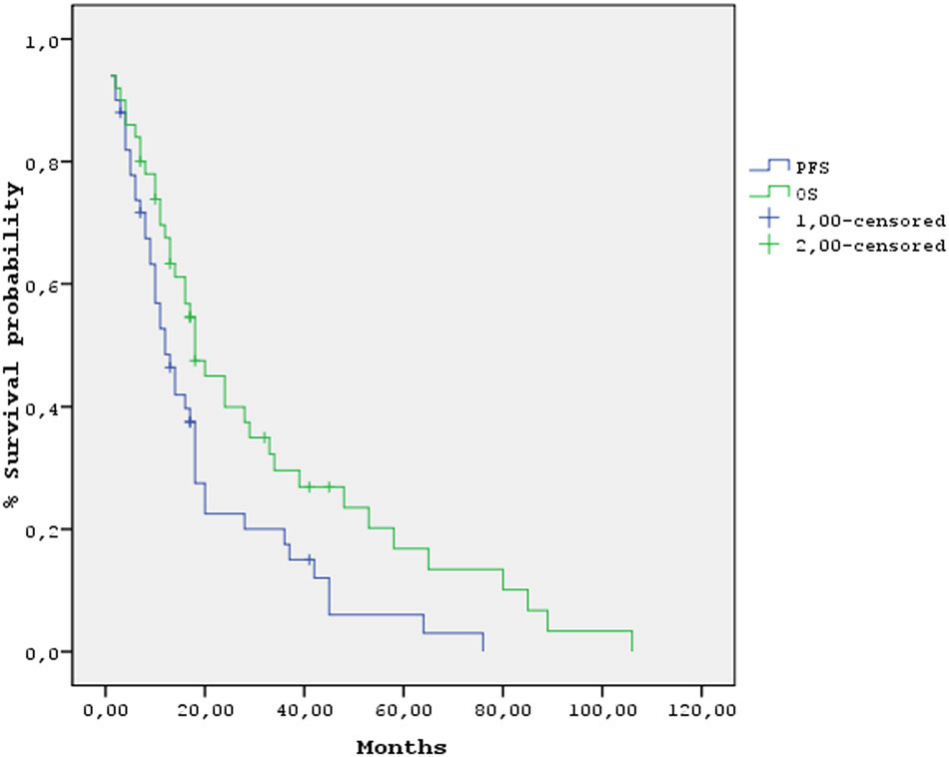
Progression-free survival (PFS) (blue curve) and overall survival (OS) (green curve) in the studied population

**Fig. 2:**
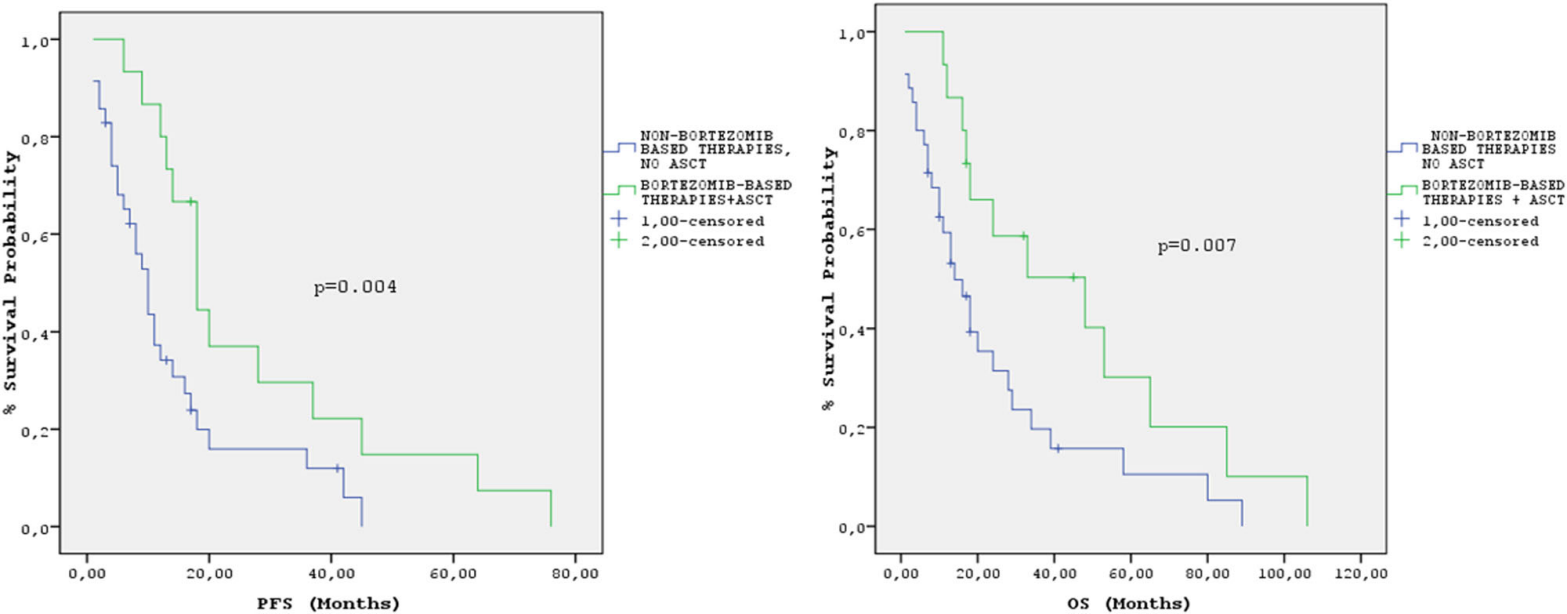
**a** Progression-free survival (PFS) in patients treated with non-bortezomib based therapies, without autologous transplantation (blue curve) and patients treated with bortezomib-based regimens and autologous transplantation (green curve). **b** Overall survival (OS) in patients treated with treatments non- bortezomib based therapies without autologous transplantation (blue curve) and patients treated with bortezomib-based regimens and autologous transplantation (green curve)

**Fig. 3:**
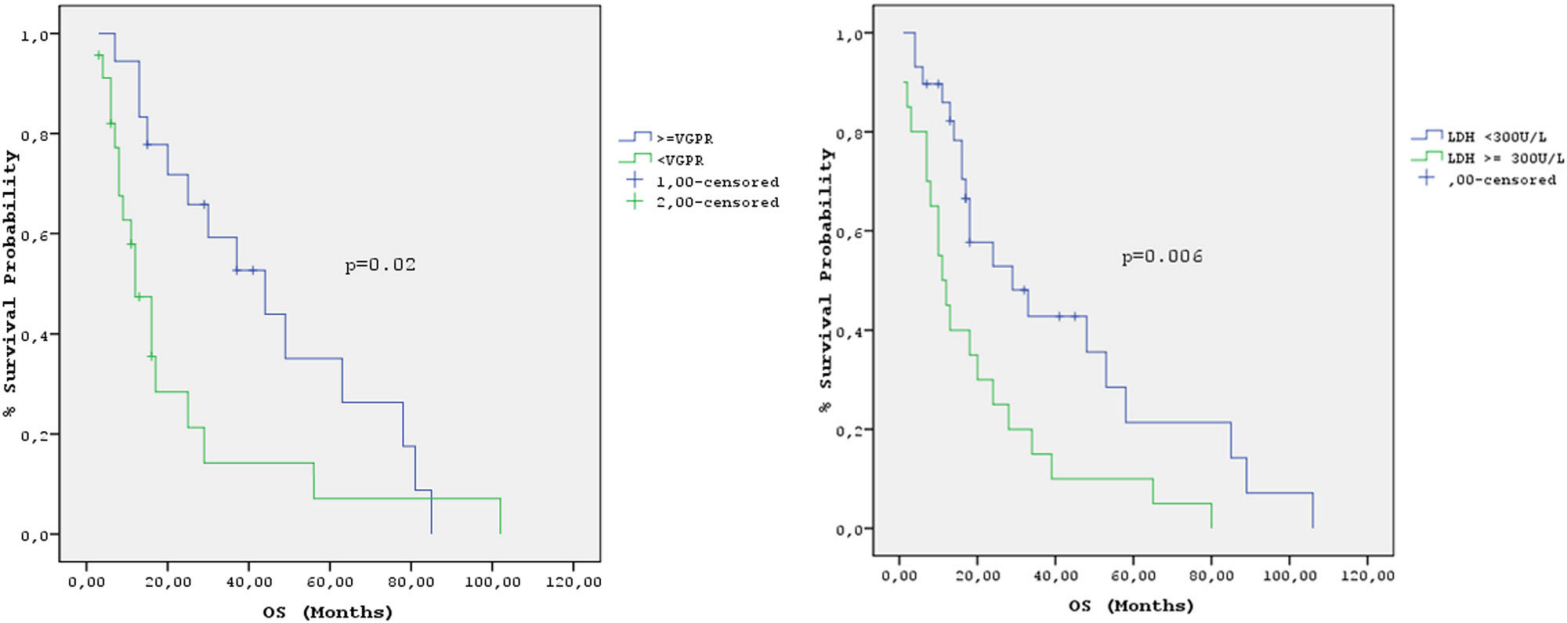
**a** Overall survival (OS) in patients who achieved at least vgPR (blue curve) and patients achieved < vgPR (green curve) (landmark analysis). **b** Overall survival (OS) in patients with LDH at baseline < 300 U/L (blue curve) and in patients with LDH ≥ 300 U/L (green curve)

Regarding the whole study population, the median PFS was 12 months (95% CI: 8.5–15.4) and the median OS was 18 months (95% CI: 14–22 months) (Fig. 1); PFS was significantly longer in patients treated with bortezomib-based therapy + ASCT vs. others (18 months, 95% CI: 13–22 months vs. 9 months, 95% CI: 6–12 months, *p* = 0.004) (Fig. 2a). Median OS was more than three times longer in patients treated with bortezomib-based regimens + ASCT vs. others (48 months 95% CI: 12–84 vs. 14 months, 95% CI: 8–20 months, *p* = 0.007) (Fig. 2b); 2-year and 3-year OS were 59 and 50%, respectively, for patients treated with bortezomib-based regimens + ASCT vs. 28 and 16%, respectively for the others. In the univariate analysis, ECOG performance status, baseline LDH ≥ 300 U/L, age ≥65, treatment with bortezomib-based regimens, ASCT, treatment with bortezomib-based regimens + ASCT and achievement of ≥vgPR predicted for OS (Table 3); LDH cut off value was used based on a previous publication of our group demonstrating that LDH ≥ 300 U/L represents a powerful prognostic factor for OS in MM patients^17^. In the multivariate analysis achievement of ≥vgPR and baseline LDH ≥ 300 U/L significantly predicted for OS (Table 3). The median OS for patients who achieved ≥vgPR was 48 months (95% CI: 23–73) vs. 13 months (95% CI: 9–17) for those achieved <vgPR (*p* = 0.003). A landmark analysis which was performed at 4 months from start of treatment confirmed the positive prognostic significance of ≥vgPR for OS; median OS for patients with ≥vgPR vs. those with <vgPR was 44 months (95% CI: 21–67) vs. 12 months (95% CI: 7–17) (*p* = 0.02, HzR: 0.45) (Fig. 3a). The median OS for patients with LDH ≥ 300 U/L was 11 months (95% CI: 7–15 months) vs. 29 months (95% CI: 9–49 months) for those with LDH < 300 U/L (*p* = 0.006) (Fig. 3b).

**Table 3:** Cox regression analysis

Variable	*p*	HR	95% CI
*Univariate cox regression analysis*			
ECOG	0.003		
AGE ≥ 65	0.02	0.49	0.26–0.92
LDH baseline ≥ 300 U/L	0.008	0.41	0.2–0.7
Bortezomib-based regimens (first line)	0.03	0.46	0.22–0.9
Bortezomib-based regimens + ASCT (first line)	0.01	0.38	0.2–0.8
ASCT	0.01	0.40	0.2–0.8
≥VGPR	0.009	0.40	0.20–0.8
*Multivariate cox regression analysis*			
LDH baseline ≥ 300 U/L	0.03	0.45	0.2–0.9
≥VGPR	0.01	0.39	0.2–0.8

*ECOG* Eastern Cooperative Oncology Group, *LDH* lactate dehydrogenase, *vgPR* very good partial response, *ASCT* autologous stem cell transplantation, *HR* hazard ratio

## Discussion

Primary plasma cell leukemia is a rare and aggressive plasma cell disorder with a very poor outcome^7^. The use of conventional therapies exhibited low response rates and a median OS ranging from 2–7 months^6–8^. In the first Surveillance, Epidemiology, and End Results (SEER) database analysis published in 2009 that evaluated characteristics and survival of 291 patients with pPCL diagnosed in the USA between 1973 and 2004^6^, the median OS was 4 months; in this analysis of pPCL patients prevalently treated with conventional chemotherapy, it was concluded that no significant OS improvement was observed over a 30-year period of observation^6^. According to studies conducted before the wide incorporation of bortezomib into first line treatment, autologous transplantation, led to a 3-year OS of 64%^9,18^. The efficacy of allogeneic transplantation was evaluated in two studies: the 3-year and 5-year OS was only 32 and 36% respectively for patients treated with a myeloablative approach, reflecting the high non-relapse mortality rate of this procedure^9,19^. Nevertheless, allogeneic transplantation is considered the only potentially curative option for subsets of young and fit patients^4,5^. The introduction of first generation novel agents, i.e., thalidomide, lenalidomide, and bortezomib has improved response rates and OS^20^ of MM patients, therefore it was reasonable to explore their role in PCL^4,5^. Novel agent-based therapies and more commonly bortezomib-based combinations were evaluated retrospectively in several case reports and case series, that included limited number of patients, as expected, considering the rarity of the disease^5,11–13^; according to those studies, objective response ranged from 56-80% and median OS ranged from 12–31 months, depending on the incorporation or not of ASCT in the induction therapy^5,11–13^. We have previously suggested that, the efficacy of bortezomib could be at least partially related to abnormal CD27 expression whose triggering on pPCL cells has a significant anti-apoptotic effect involving ERK1/2, NF-kB, and JNK signal transduction pathways^21^. The majority of studies showed survival advantage in patients treated with bortezomib-based combinations compared to those treated with conventional therapy^11–13^ however, the number of patients with pPCL included in those studies was limited. At variance, in the study performed by the Intergroup Francophone du Myelome (IFM)^14^ there was no survival advantage with the use bortezomib-based combinations, however, the exact number of patients treated with bortezomib, conventional treatments or thalidomide-based regimens was not reported^14^. Likewise, in the analysis of 27 patients with pPCL reported by Usmani and colleagues^15^, the addition of bortezomib in the total therapy programs did not improve OS of patients with pPCL as this was the case for MM patients^15^. In the recently published updated analysis of the SEER database, which is currently the largest timeless published analysis of pPCL a significant OS improvement was observed in patients treated after 2006 compared to those treated before 2006^10^. Despite the lack of specific information concerning the types of therapy, it is most possible that the wide use of novel agents after 2006 in this setting was the main reason for the observed survival improvement^10^. Recently, two prospective studies evaluated the efficacy of bortezomib-based combinations^16^ or Lenalidomide-Dexamethasone (Len-Dex)^21^ in pPCL. The first study conducted by the IFM demonstrated that bortezomib-based regimens followed by ASCT exhibit a 69% objective response rate and a median OS of 36 months^16^. In the second study conducted by the Italian group^22^, 23 patients with pPCL received the Len-Dex combination; ORR was 74% and the median OS was 28 months, suggesting that LenDex combination could be a reasonable option, particularly for elderly patients; however, taking into account that LenDex has been recently approved as first line therapy in transplant ineligible patients, there are no data regarding the efficacy of the combination in pPCL, in the real-world setting. In the current study, we presented real-world data on prognosis and outcome of one of the largest reported to-date series of patients with pPCL most of whom were treated with bortezomib-based combinations with or without ASCT, outside the context of clinical trials. Objective response and at least vgPR was achieved in 76 and 38%, respectively, in the whole population, reaching 100 and 73%, respectively, in those treated with bortezomib-based combinations plus ASCT; furthermore, the achievement of sustained objective response, i.e., lasting for at least 1 year strongly correlated with bortezomib-based induction followed by ASCT, suggesting that this is probably the optimum current treatment approach among approved therapies in the first line setting, as recommended by the International myeloma Working Group (IMWG)^4^. Considering that published data regarding prognostication of pPCL are limited, the main goal of the current study was to look for significant prognostic factors for OS. We have demonstrated that bortezomib-based combinations plus ASCT upfront, strongly correlated with OS exhibiting a 62% reduction in the probability of death. The prognostic significance of treatment with bortezomib-based combinations plus ASCT was not maintained in the multivariate analysis even after omitting from the analysis age and performance status which could be likely confounders (data not shown); achievement of at least vgPR proved to be more powerful, indicating that quality response is mandatory for the improvement of disease outcome, regardless of how this is obtained. Nevertheless, achievement of at least vgPR strongly correlated with bortezomib-based combinations plus ASCT highlighting the positive predictive role of this treatment approach in pPCL.

We would like to commend that patients did not receive lenalidomide-dexamethasone (LenDex) or lenalidomide-dexamethasone-bortezomib (VRD) at first line, as lenalidomide was not approved in Greece until recently. However, an appreciable proportion of patients received lenalidomide-based therapies including LenDex or VRD beyond first line, as well as triplet combinations of next generation novel agents such as carfilzomib, pomalidomide, ixazomib and daratumumab. Of note, salvage therapy with novel agents, did not correlate with OS or post-relapse survival in patients treated upfront with either bortezomib-based combinations or those treated with non-bortezomib based therapies, indicating that the poor outcome of the latter group correlated with the omission of novel agents in first line therapy rather than in relapse; moreover, only 5/12 patients (41%) treated with conventional approach, reached second line therapy compared to 66% of those treated with bortezomib-based therapies; taking together these observations, support the idea of offering the most effective available treatments upfront, to induce deep and durable responses that lead to suppression of resistant clones which are commonly present at the time of diagnosis especially in aggressive forms of plasma cell dyscrasias, such as pPCL or cytogenetically defined high risk MM^23–25^. Given the fact that maintenance therapy has not been the standard of care in Greece at least until recently, maintenance with LenDex was offered only in two patients treated upfront with bortezomib-based combinations followed by ASCT (data not shown); interestingly, those patients were the only patients who achieved a PFS of more than 5 years; this observation underscores the possible role of continuous therapy in pPCL^5^. Regarding early mortality (EM) rate, it was very low (6%) in our study and it was significantly correlated with induction treatments that did not include bortezomib. In the updated SEER database analysis, the reported EM rate before 2006 was 26 vs. 15% after 2006, probably reflect, the efficacy of novel agents, most of which were available for use in the upfront setting after 2006. With regard to safety, the current study confirmed our previous observations^11^ that bortezomib-related toxicity is limited and manageable. Interestingly, grade 3/4 neurotoxicity was very low, in accordance with the IFM study in which there was zero grade 3/4 neurotoxicity. The low incidence of severe peripheral neuropathy reflects the improvement in the management of bortezomib-related neuropathy, as physicians have gained experience over time.

In conclusion, we have confirmed in a large population of unselected patients with exclusively primary PCL that bortezomib-based regimens exhibit high efficacy and significantly reduce early mortality rate. Moreover, we have shown that bortezomib-based therapies plus ASCT consolidation, is currently the best available therapeutic approach for the treatment of pPCL upfront, providing deep and durable responses that translate into prolonged OS. It is reasonable to speculate that next generation therapies, such as novel proteasome inhibitors and monoclonal antibodies, which have shown efficacy in cytogenetically defined high-risk MM, may also exhibit high efficacy in pPCL; however, so far there are no published data on the efficacy of these drugs in pPCL, as such patients are excluded from prospective studies. Therefore, the efficacy of next generation novel agents remains to be seen in well-designed prospective studies. Currently, the European Myeloma network is conducting a study in pPCL (EMN12/HOVON129 PCL) in which patients will be treated with carfilzomib, lenalidomide, and dexamethasone in induction, consolidation, and maintenance. In addition, younger patients will receive the tandem of ASCT and allo-SCT or, in case of no suitable donor, tandem ASCT. The design of the latter study which is based on using treatments with novel drugs in a continuous fashion, underscores the importance of treating pPCL timely and effectively following in some way the therapeutic approach of acute leukemia.
